# Supplementary material for: Preclinical Development of a Vectorized Artificial miRNA Gene Therapy for Tauopathies
Source: bioRxiv. 2025 Oct 14:2025.10.12.681935. Preprint. [Version 1] doi: 10.1101/2025.10.12.681935 (PMC12633006; doi:10.1101/2025.10.12.681935)
Supplement: Supplement 2 [file NIHPP2025.10.12.681935v1-supplement-2.pdf]

bioRxiv preprint doi: https://doi.org/10.1101/2025.10.12.681935; this version posted October 14, 2025. The copyright holder for this preprint (which was not certified by peer review) is the author/funder, who has granted bioRxiv a license to display the preprint in perpetuity. It is made available under aCC-BY 4.0 International license.

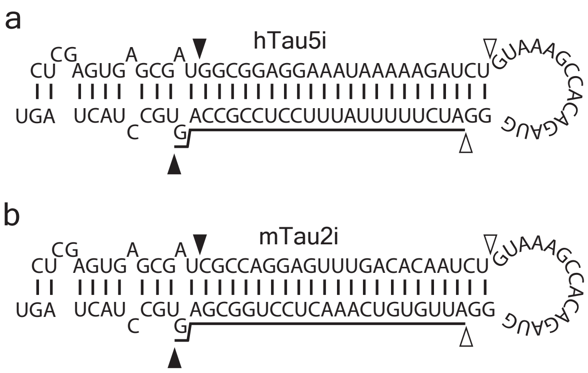

Supplementary Figure 1. In silico prediction of artificial tau miRNAs. a,b UNAFOLD hairpin 2 dimensional structures and sequences of lead human **(a)** and mouse **(b)** artificial miRNAs. Black and gray arrowheads indicate Drosha and Dicer cut sites respectively in the modified human miR-30a cassette and the underlined area indicates the guide miRNA strand sequence.

bioRxiv preprint doi: https://doi.org/10.1101/2025.10.12.681935; this version posted October 14, 2025. The copyright holder for this preprint (which was not certified by peer review) is the author/funder, who has granted bioRxiv a license to display the preprint in perpetuity. It is made available under aCC-BY 4.0 International license.

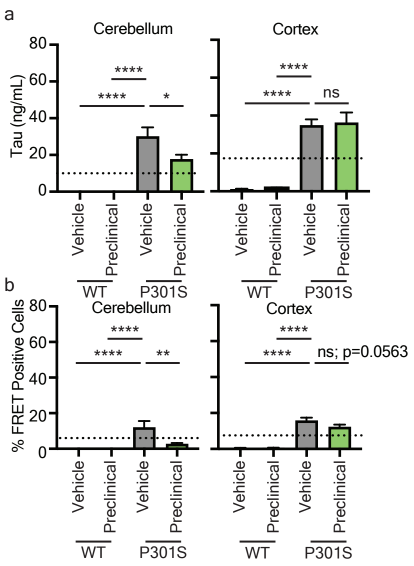

Supplementary Figure 2. 3-month efficacy data. a ELISA quantification of 1N4R human P301S tau cerebellum and cortex; **b** Soluble tau seeding of cerebellum and cortex lysates from wildtype and P301S littermates using HEK293T tau biosensors. Mean±SD; One-way ANOVA with Tukey's multiple comparisons; * p< 0.05; ** p< 0.01; ****p<0.0001. Dashed lines in histoplots represent the half maximal inhibitory value relative to the control group.

bioRxiv preprint doi: https://doi.org/10.1101/2025.10.12.681935; this version posted October 14, 2025. The copyright holder for this preprint (which was not certified by peer review) is the author/funder, who has granted bioRxiv a license to display the preprint in perpetuity. It is made available under aCC-BY 4.0 International license.

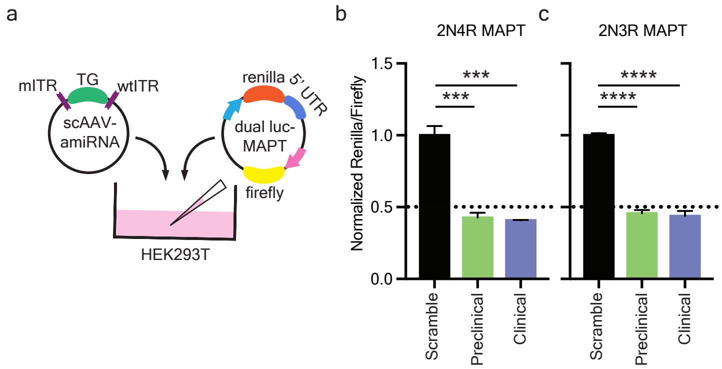

Supplementary Figure 3. Preclinical and clinical vector designs display comparable total tau gene reduction. a Illustration of experimental paradigm of the self-complimentary AAV constructs co-transfected into microwell plates with the MAPT dual luciferase screening constructs; **b,c** Histoplots illustrate the reduction of human 2N4R **(b)** and 2N3R **(c)** MAPT isoform knockdown using a dual luciferase reporter system. Mean±SD; One-way ANOVA with Tukey's multiple comparisons; *** $p < 0.001$; ****$p < 0.0001$. Dashed lines in histoplots represent the half maximal inhibitory value relative to the control group.

bioRxiv preprint doi: https://doi.org/10.1101/2025.10.12.681935; this version posted October 14, 2025. The copyright holder for this preprint (which was not certified by peer review) is the author/funder, who has granted bioRxiv a license to display the preprint in perpetuity. It is made available under aCC-BY 4.0 International license.

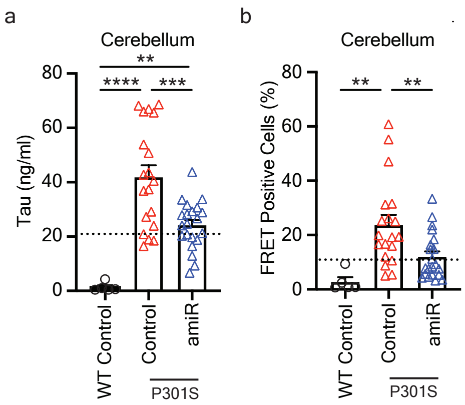

Supplementary Figure 4. 6-month efficacy data. a ELISA Quantification of 1N4R human P301S tau protein in the cerebellum of wildtype and P301S injected littermates; **b** Quantification of soluble tau seeding from the cerebellum. Mean±SD; One-way ANOVA with Tukey's multiple comparisons; ** $p < 0.01$; ***$p<0.001$; ****$p<0.0001$. Dashed lines in histoplots represent the half maximal inhibitory value relative to the control group.

bioRxiv preprint doi: https://doi.org/10.1101/2025.10.12.681935; this version posted October 14, 2025. The copyright holder for this preprint (which was not certified by peer review) is the author/funder, who has granted bioRxiv a license to display the preprint in perpetuity. It is made available under aCC-BY 4.0 International license.

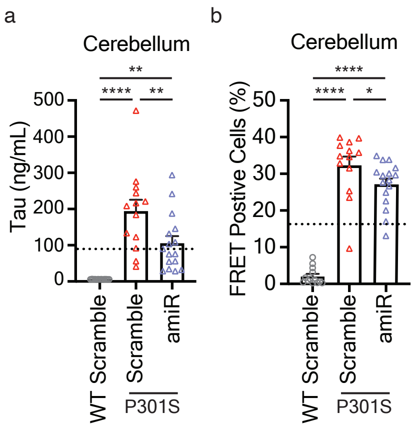

Supplementary Figure 5. 9-month efficacy data. a,b Histoplots illustrate tau protein **(a)** quantification by ELISA and tau seeding **(b)** from cerebellum of wildtype and P301S injected littermates. Mean±SEM; One-way ANOVA with Tukey's multiple comparisons; * p< 0.05; ** p< 0.01; ****p<0.0001. Dashed lines in histoplots represent the half maximal inhibitory value relative to the control group.

bioRxiv preprint doi: https://doi.org/10.1101/2025.10.12.681935; this version posted October 14, 2025. The copyright holder for this preprint (which was not certified by peer review) is the author/funder, who has granted bioRxiv a license to display the preprint in perpetuity. It is made available under aCC-BY 4.0 International license.

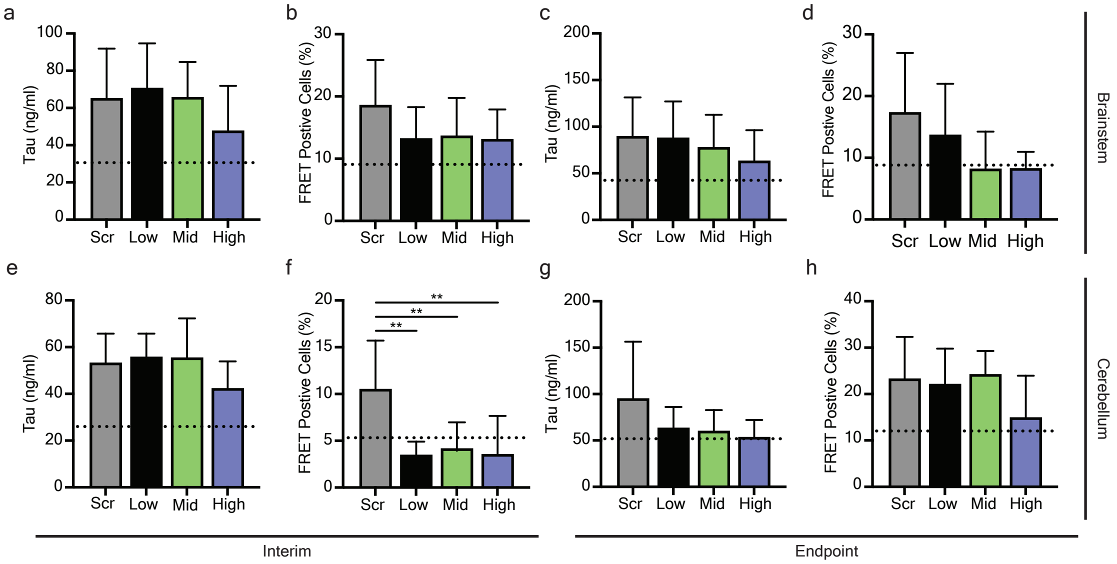

Supplementary Figure 6. Dosing biochemical data. a-h Histoplots illustrate quantification of brainstem **(a-d)** and cerebellum **(e-h)** samples from interim **(a,b,e,f)** or endpoint **(c,d,g,h)** animal's tau protein **(a,c,e,g)** and tau seeding **(b,d,f,h)**. Mean±SEM; One-way ANOVA with Tukey's multiple comparisons; ** $p < 0.01$. Dashed lines in histoplots represent the half maximal inhibitory value relative to the control group.
